# Harnessing Induced Essentiality: Targeting Carbonic Anhydrase IX and Angiogenesis Reduces Lung Metastasis of Triple Negative Breast Cancer Xenografts

**DOI:** 10.3390/cancers11071002

**Published:** 2019-07-17

**Authors:** Eva-Maria E. Hedlund, Paul C. McDonald, Oksana Nemirovsky, Shannon Awrey, Lasse D.E. Jensen, Shoukat Dedhar

**Affiliations:** 1 Department of Integrative Oncology, BC Cancer Research Centre, Vancouver, BC V5Z 1L3, Canada; 2 Department of Medical and Health Sciences (IMH), Division of Cardiovascular Medicine (KVM), Linköping University, SE-581 85 Linköping, Sweden; 3 Department of Biochemistry and Molecular Biology, University of British Columbia, Vancouver, BC V6T 1Z3, Canada

**Keywords:** angiogenesis, carbonic anhydrase IX, hypoxia, metastasis, resistance, sunitinib, triple negative breast cancer

## Abstract

Triple Negative Breast Cancer (TNBC) is aggressive, metastatic and drug-resistant, limiting the spectrum of effective therapeutic options for breast cancer patients. To date, anti-angiogenic agents have had limited success in the treatment of systemic breast cancer, possibly due to the exacerbation of tumor hypoxia and increased metastasis. Hypoxia drives increased expression of downstream effectors, including Carbonic Anhydrase IX (CAIX), a critical functional component of the pro-survival machinery required by hypoxic tumor cells. Here, we used the highly metastatic, CAIX-positive MDA-MB-231 LM2-4 orthotopic model of TNBC to investigate whether combinatorial targeting of CAIX and angiogenesis impacts tumor growth and metastasis in vivo to improve efficacy. The administration of a small molecule inhibitor of CAIX, SLC-0111, significantly reduced overall metastatic burden, whereas exposure to sunitinib increased hypoxia and CAIX expression in primary tumors, and failed to inhibit metastasis. The administration of SLC-0111 significantly decreased primary tumor vascular density and permeability, and reduced metastasis to the lung and liver. Furthermore, combining sunitinib and SLC-0111 significantly reduced both primary tumor growth and sunitinib-induced metastasis to the lung. Our findings suggest that targeting angiogenesis and hypoxia effectors in combination holds promise as a novel rational strategy for the effective treatment of patients with TNBC.

## 1. Introduction

Breast cancer is composed of several distinct subtypes that exhibit markedly different outcomes [1,2]. Triple negative breast cancer (TNBC) is particularly aggressive and is often resistant to treatment [3,4]. Furthermore, TNBC is prone to metastasis, a major contributor to tumor recurrence and cancer-associated mortality. These features highlight a critical need for the development of novel therapeutic strategies that effectively target this malignancy.

Amongst available therapeutic options, anti-angiogenic agents have been reported to inhibit breast tumor growth [5]. However, as a consequence of their activity, these agents can exacerbate tumor hypoxia, leading to disease recurrence, metastasis and death [5,6]. Acquired resistance by tumor cells, for example, adaptation to survive in hypoxia, is linked to the failure of anti-angiogenic agents as monotherapy [7]. Although initial Food and Drug Administration (FDA) approval of bevacizumab for metastatic breast cancer was subsequently rescinded [8], anti-angiogenic agents remain in use for the treatment of breast cancer in many jurisdictions, including Europe [9], and studies have demonstrated increased efficacy when used in combination with chemotherapy [10,11]. Furthermore, recent meta-analyses have shown a significant clinical benefit of anti-angiogenic agents in combination with chemotherapy in the neoadjuvant setting for Human Epidermal Growth Factor Receptor 2 (HER2)-negative breast cancer patients [9,12], as well as for maintenance therapy in metastatic disease [13]. This evolving treatment landscape provides a unique opportunity to combine anti-angiogenic agents and agents that target hypoxia effectors with the goal of enhancing the response to therapy.

In response to hypoxia, tumor cells undergo a metabolic switch and, through the activation of Hypoxia Inducible Factor 1 alpha (HIF1α), shift rapidly toward increased glycolysis and glutaminolysis, and reduced oxidative phosphorylation, for energy production and macromolecular biosynthesis [14]. The acidic metabolic end products, including lactate, protons (H^+^) and carbon dioxide (CO_2_), contribute to the perturbation of intracellular pH (pHi) homeostasis, a situation with potentially lethal consequences [15]. To survive hypoxic and acidic cellular stress, tumor cells invoke a complex pH regulatory system geared toward the maintenance of pHi homeostasis. One important component of this system is Carbonic Anhydrase IX (CAIX), a metabolic transmembrane enzyme with an extracellular facing catalytic domain that regulates the reversible hydration of carbon dioxide (CO_2_) to bicarbonate (HCO_3_^−^) and H^+^ [16]. In doing so, CAIX contributes both to the regulation of pHi and to the acidification of the tumor microenvironment, promoting tumor cell invasion and metastasis [17,18].

CAIX is overexpressed in breast cancer, is particularly highly expressed in TNBC, and is an established biomarker of poor prognosis [17,19]. Studies have demonstrated that CAIX is a critical regulator of hypoxia-mediated breast tumor growth and metastasis [19,20], and evidence suggests that CAIX is important for the maintenance of breast cancer stem cells (CSCs) within the hypoxic niche [21]. Such findings have driven the development of novel small molecule inhibitors of CAIX, and studies have shown that these inhibitors are effective at reducing breast tumor growth and metastasis [19,22,23]. Furthermore, recent studies have demonstrated that targeting CAIX in combination with chemotherapy or immune checkpoint blockade increases efficacy in several pre-clinical tumor models, including pancreatic cancer, glioblastoma, melanoma and TNBC [24,25,26].

Since hypoxia induced by anti-angiogenic therapy may stimulate resistance-promoting adaptations—including the expression of CAIX—by tumor cells, targeting these effectors in combination with inhibitors of angiogenesis may induce a specific form of synthetic lethality called induced essentiality (aka contextual synthetic lethality) [27,28]. Indeed, studies have shown that genetic depletion of CAIX expression enhances the efficacy of bevacizumab in preclinical tumor models [29]. Furthermore, recent evaluation of soluble CAIX (sCAIX) induced by shedding in breast cancer patients treated with bevacizumab showed that sCAIX levels are predictive of therapeutic response to inhibition of angiogenesis [30]. However, the impact of targeting CAIX activity in combination with anti-angiogenic agents in a therapeutically relevant manner in breast cancer has not been evaluated.

Here, we used the highly metastatic MDA-MB-231 LM2-4 orthotopic model of TNBC, a prototypic model for studying human breast cancer metastasis in vivo [10,11], to investigate the effect of pharmacologically targeting CAIX activity and angiogenesis, alone and in combination, on tumor growth and metastasis. We found that the administration of sunitinib to mice with orthotopic breast cancer xenografts significantly inhibited tumor growth, but failed to reduce metastases. In sharp contrast, the administration of a clinically-validated inhibitor of CAIX activity, SLC-0111, significantly reduced spontaneous metastasis. Exposure to sunitinib increased hypoxia and CAIX expression in primary tumors, while the administration of SLC-0111 resulted in reduced permeability of the primary tumor vasculature, as well as reduced lung and liver metastasis. Furthermore, the administration of sunitinib and SLC-0111 in combination significantly inhibited sunitinib-induced metastasis to the lung, suggesting the potential capacity of the combination to enhance efficacy for patients with TNBC.

## 2. Results

### 2.1. Targeting CAIX Activity and Angiogenesis Reduces Tumor Growth and Metastasis in an Orthotopic Xenograft Model of TNBC

**Figure 1:**
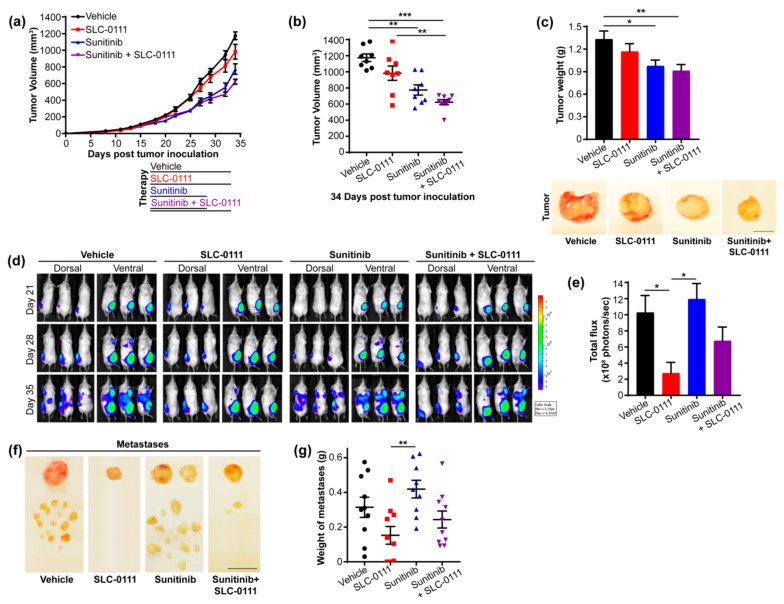
Pharmacologic inhibition of Carbonic Anhydrase IX (CAIX) activity and angiogenesis decrease tumor growth and metastasis in an in vivo model of Triple Negative Breast Cancer (TNBC). Mice with orthotopic MDA-MB-231 LM2-4Luc^+^ tumors were given SLC-0111 (50 mg/kg) and sunitinib (60 mg/kg), alone or in combination, daily by oral gavage. (**a**) Tumor growth curve. The schematic indicates the treatment schedule. For the combination, mice received sunitinib and SLC-0111 simultaneously from day 17 to day 27 post tumor cell inoculation, followed by SLC-0111 alone until endpoint. Data show the mean ± standard error of the mean (SEM). *n* = 8–10 animals/group. (**b**) Analysis of tumor volume at the tumor growth endpoint. Data show the mean ± SEM. *n* = 8–9 animals/group. ** *p* < 0.01, *** *p* < 0.001. (**c**) Analysis of tumor weight. Data show the mean ± SEM. *n* = 9 animals/group. * *p* < 0.05, ** *p* < 0.01. Representative images of primary tumors are shown below the graph. Scale bar = 1 cm. (**d**) Bioluminescence imaging (BLI) showing the change in metastatic burden with time. Representative images of animals from each group are shown. Heat map indicates increasing BLI intensity from low (blue) to high (red). (**e**) Quantification of BLI-derived metastatic burden at day 35 post tumor inoculation. Data show the mean ± SEM. *n* = 7–10 animals/group. * *p* < 0.05. (**f**) Images showing metastatic nodules harvested from animals in each group. Each image shows axillary and/or inguinal lymph node metastases (top) together with grossly resectable metastatic foci present within the abdominal cavity (bottom) of 1 representative animal/group. Scale bar = 1 cm. (**g**) Quantification of the weight of metastases harvested from animals described in panel f. Data show the mean ± SEM. *n* = 9–10 animals/group. Number of resectable metastatic foci/animal = 0–32. ** *p* < 0.01.

To explore whether targeting CAIX activity in the context of anti-angiogenic agents may improve efficacy in the setting of TNBC in vivo, we focused our studies on the highly metastatic, CAIX-positive, MDA-MB-231 LM2-4Luc^+^ orthotopic human breast tumor model [5]. Tumors were established and animals were given SLC-0111 and sunitinib, either alone or in combination. Daily administration of 60 mg/kg sunitinib for 11 days, a dose and duration previously reported to provide tumor growth inhibition while mitigating toxicity and emulating a clinically relevant dosing schedule [5], significantly inhibited primary tumor growth when compared to vehicle controls (Figure 1a,b). The concurrent administration of SLC-0111 and sunitinib further reduced tumor growth, with significant differences relative to vehicle and SLC-0111 (Figure 1a,b). Similarly, the administration of sunitinib, both alone and in combination with SLC-0111, significantly reduced tumor weight (Figure 1c) and inhibited cell proliferation, as shown by immunohistochemical staining for Ki67 (Figure S1), confirming the effect of drug exposure on tumor growth. Sunitinb and SLC-0111 were well-tolerated and did not result in significant weight loss during administration (Figure S2).

Next, we assessed the impact of SLC-0111 and sunitinib on overall metastatic burden. Mice with MDA-MB-231 LM2-4Luc^+^ tumors were administered the agents as described above and were monitored for metastases longitudinally using bioluminescence imaging (BLI) (Figure 1d). Dorsal and ventral images were acquired for all animals at each time point to facilitate capture of the full spectrum of metastasis. Due to the highly aggressive disease course exhibited by this model, primary tumors were not resected, and the bioluminescence signals from both the primary tumor and the metastases are visible in the images. Primary tumors were visible in all groups at day 21 post tumor inoculation (Figure 1d). Metastases were evident in animals given vehicle or sunitinib, but not those administered SLC-0111, at day 28 post tumor inoculation (Figure 1d). At day 35 of the study, mice administered vehicle or sunitinb showed extensive metastatic burden. In contrast, markedly reduced metastasis was observed in animals given SLC-0111 alone or in combination with sunitinib (Figure 1d). To quantitatively evaluate the metastatic burden in the mice, the bioluminescence imparted by the primary tumors was subtracted from the total bioluminescence detected in each animal to derive the signal contributed by the metastases. Quantification of luminescence derived from the metastases revealed a dramatic and significant reduction in metastatic burden in mice provided SLC-0111 compared to animals administered sunitinb or vehicle and showed a trend toward reduced metastasis in animals administered combination therapy (Figure 1e). Interestingly, animals given sunitinib did experience significant weight loss near the end of the study, likely reflecting increased disease burden, while animals given SLC-0111 alone or in combination with sunitinib maintained stable weights (Figure S2).

At necropsy, grossly visible metastastic foci present in the axillary and inguinal lymph nodes, the abdominal cavity, and subcutaneous regions were resected, photographed, and weighed. Evaluation of metastatic burden of individual mice revealed a near complete absence of metastasis in 4 of the 10 animals to which SLC-0111 had been administered (Figure 1f,g). In contrast, while animals given sunitinib showed significantly reduced primary tumor growth (Figure 1a), these animals exhibited enhanced metastatic burden (Figure 1f,g), and the administration of SLC-0111 in combination with sunitinib reduced the overall metastatic burden in this cohort, although statistical significance was not reached (Figure 1f,g).

### 2.2. Sunitinib Induces Hypoxia and CAIX Expression in Primary Breast Tumors

**Figure 2:**
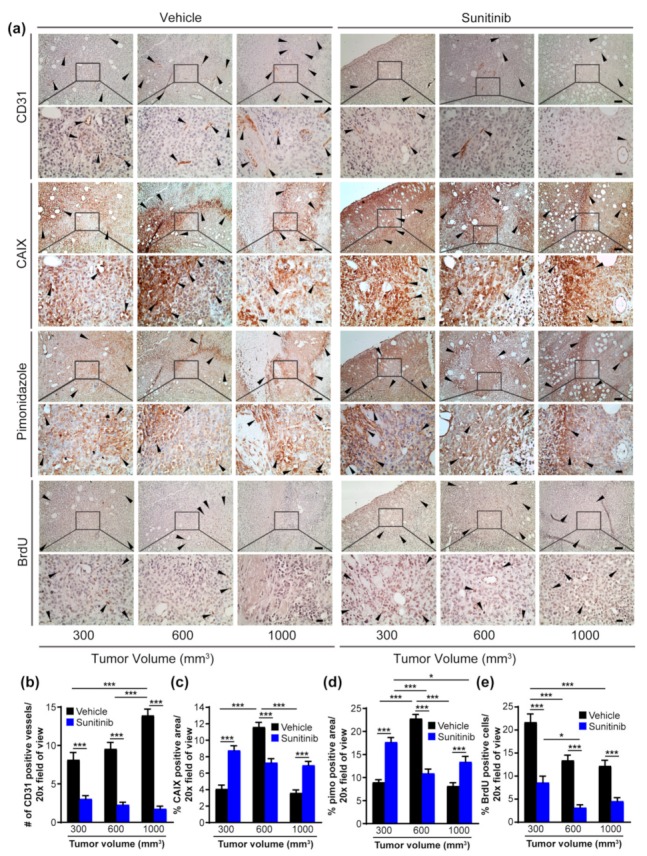
Sunitinib induces hypoxia and Carbonic Anhydrase IX (CAIX) expression in primary Triple Negative Breast Cancer (TNBC) tumors. (**a**) Images of MDA-MB-231 LM2-4Luc^+^ primary tumor tissue sections harvested at increasing tumor volumes from animals administered either vehicle or 60 mg/kg sunitinib and immunohistochemically stained for the indicated markers. Scale bars: upper panels, 1 mm; lower panels, 200 μm. (**b** to **e**) Image-based quantification of (**b**) CD31^+^ blood vessels, (**c**) CAIX expression, (**d**) pimonidazole and (**e**) proliferation. Data show the mean ± standard error of the mean (SEM). *n* = 3 animals/group with 10 images/animal. * *p* < 0.05, *** *p* < 0.001.

To evaluate the impact of anti-angiogenic agents on the temporal evolution of hypoxia in the TME of TNBC, we administered either sunitinib or vehicle to mice with orthotopic MDA-MB-231 LM2-4Luc^+^ tumors, collected a subset of animals from each group as tumor sizes reached pre-defined target volumes of approximately 300, 600 and 1000 mm^3^, and analyzed the tumors by immunohistochemistry (IHC) for markers of hypoxia and proliferation. Compared to control tumors, which showed increased numbers of CD31^+^ blood vessels at large tumor volumes, significantly fewer vessels were evident in tumors exposed to sunitinib at tumor volumes of 300 mm^3^ and vessel density did not increase with larger tumor sizes but, in fact, decreased (Figure 2a,b), indicating response to sunitinib.

Next, we determined whether CAIX expression by tumor cells, considered an endogenous biomarker of hypoxia in breast cancer [31], was impacted as a consequence of reduced angiogenesis in tumors exposed to sunitinib. Compared to levels of CAIX expression in control tumors, which increased significantly as tumors grew from 300 to 600 mm^3^, and declined as tumors became more vascularized, tumors from mice administered sunitinib demonstrated significantly higher levels of CAIX expression early during tumor progression, and levels remained consistently elevated at larger volumes (Figure 2a,c), consistent with increased hypoxia resulting from diminished vasculature. Staining for pimonidazole (pimo), an established marker of hypoxia [32], in adjacent tissue sections mirrored the levels of expression of CAIX (Figure 2a,d), confirming that sunitinib results in increased hypoxia and a concomitant increase in the levels of expression of CAIX in both the early and later stages of breast tumor growth.

To examine the proliferative capacity of these tumors, tissue sections were stained for 5-bromo-2’-deoxyuridine (BrdU). In tumors from animals administered vehicle, the number of BrdU positive cells was largest in the small tumors and decreased significantly with tumor size (Figure 2a,e). However, tumors from mice given sunitinib demonstrated significantly lower numbers of BrdU positive cells at all tumor volumes, indicating that these cells proliferate at a slower rate (Figure 2a,e).

### 2.3. SLC-0111 Reduces Vascular Density, Permeability and Metastasis to the Lungs and Liver

**Figure 3:**
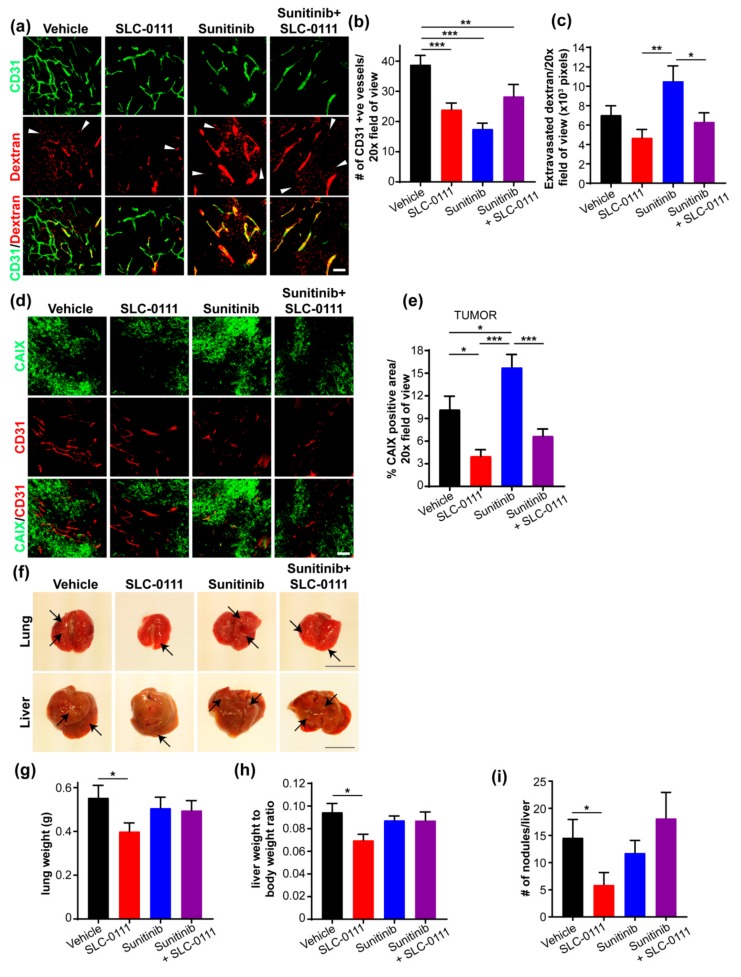
SLC-0111 reduces vessel density and vascular permeability in primary tumors, and decreases lung and liver metastases. (**a**) Representative 3D maximum projections of whole mount primary tumor tissue slices showing CD31^+^ blood vessels (green) and fluorescently labeled dextran (red). Merged images demonstrate the presence of both intravascular (yellow) and extravasated (arrowheads) dextran. Scale bar = 100 μm. (**b**) Quantification of the number of vessels in whole mount primary tumor slices. Data show the mean ± standard error of the mean (SEM). *n* = 13–14 images/group. ** *p* < 0.01, *** *p* < 0.001. (**c**) Quantification of vascular permeability as assessed by relative area of extravasated dextran in whole mount primary tumor slices. Data show the mean ± SEM. *n* = 13–16 images/group. * *p* < 0.05, ** *p* < 0.01. (**d**) Representative 3D maximum projections of whole mount primary tumor tissue slices showing levels of CAIX expression (green) and the number of CD31^+^ blood vessels (red). Lower panels, merge. Scale bar = 100 μm. (**e**) Quantification of Carbonic Anhydrase IX (CAIX) expression in whole mount primary tumor tissue slices in panel d. Data show the mean ± SEM. *n* = 11–15 images/group. * *p* < 0.05, *** *p* < 0.001. (**f**) Representative images of lung and liver tissues from animals with MDA-MB-231 LM2-4Luc^+^ orthotopic breast tumors and administered SLC-0111 and sunitinib, either alone or in combination. Visible metastatic nodules (arrows) are indicated. Scale bar = 1 cm. (**g**–**i**) Analysis of (**g**) lung weight (*n* = 9/group), (**h**) liver weight as a function of whole animal body weight (*n* = 9/group) and (**i**) number of metastatic nodules present on the liver surface (*n* = 7–8/group). For each graph, data show the mean ± SEM. * *p* < 0.05.

To examine in more detail the differences in vessel density and/or changes in vascular permeability in the primary tumor in response to SLC-0111 and sunitinib, we examined whole mount tumor tissue slices, which enable the visualization of vascular structures with increased tissue depth compared to standard IHC, for CD31 expression and the distribution of rhodamine-labeled dextran by immunofluorescence labeling coupled with 3D confocal microscopy. As expected, the number of CD31^+^ vessels in tumors from animals given sunitinib was significantly decreased compared to controls (Figure 3a,b). Interestingly, the administration of SLC-0111 also significantly reduced the density of the tumor vasculature (Figure 3a,b). Furthermore, the level of extravasated dextran was significantly reduced in animals given SLC-0111 compared to the group administered sunitinib (Figure 3a,c). Sunitinib and SLC-0111 in combination also significantly reduced the levels of dextran extravasation compared to sunitinib alone (Figure 3c). Finally, an analysis of conditioned media from MDA-MB-231 LM2-4Luc^+^ cells stably depleted of CAIX expression using short hairpin RNA (shRNA) (Figure S3a) and cultured in hypoxia demonstrated the presence of reduced levels of several angiogenic factors (Figure S3b). Validation of these results showed that Vascular Endothelial Growth Factor A (VEGFA) expression was significantly reduced in CAIX-depleted cells (Figure S3c). Collectively, these data show that the administration of SLC-0111 not only reduces the number of tumor vessels, but also reduces vascular permeability, potentially through modulation of expression and secretion of VEGFA.

**Figure 4:**
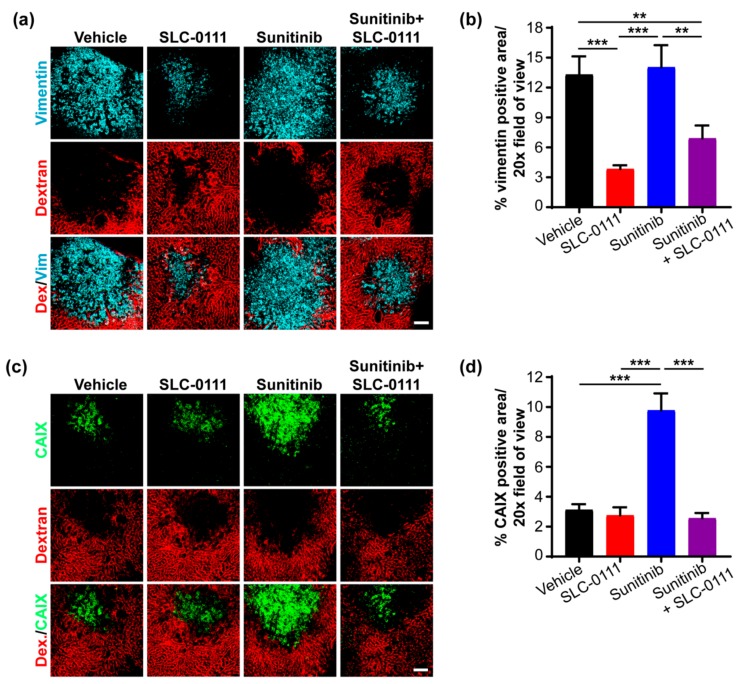
SLC-0111 inhibits sunitinib-induced Carbonic Anhydrase IX (CAIX) expression in liver metastases. (**a**) Representative 3D maximum projections of whole mount liver tissue slices from animals administered agents as indicated, showing rhodamine-labeled dextran (red) and vimentin-positive metastases (cyan). Scale bar = 100 μm. (**b**) Analysis of vimentin expression in the whole mount liver tissue sections described in panel a. Data show the mean ± standard error of the mean (SEM). *n* = 9–12 images/group: ** *p* < 0.01, *** *p* < 0.001. (**c**) Representative 3D maximum projections of whole mount liver tissue slices from animals administered agents as indicated, showing rhodamine-labeled dextran (red) and levels of expression of CAIX (green). Scale bar = 100 μm. (**d**) Analysis of CAIX expression in whole mount liver tissue sections described in panel c. Data show the mean ± SEM. *n* = 9–12 mages/group. *** *p* < 0.001.

To confirm the relationship between CAIX expression and blood vessel density in primary tumors in response to exposure to SLC-0111 and sunitinib, we co-stained whole mount tissue preparations for CAIX and CD31. SLC-0111 significantly reduced levels of CAIX expression when compared to vehicle controls (Figure 3d,e), while sunitinib significantly elevated levels of CAIX expression, relative to the levels observed in either vehicle- or SLC-0111-treated tumors (Figure 4d,e). Furthermore, the combination of sunitinib and SLC-0111 also reduced CAIX expression, concomitant with reduced vessel density (Figure 3d,e).

To directly assess metastatic burden in the lungs and liver, tumor-bearing animals were administered SLC-0111 and sunitinib, and these organs were photographed, weighed, and analyzed for the number of grossly visible nodules. Compared to controls, the administration of SLC-0111 reduced the appearance and severity of strongly visible metastatic nodules (Figure 3f, Table S1), and significantly reduced lung weight (Figure 3g), liver weight (Figure 3h) and number of liver nodules (Figure 3i). In contrast, sunitinib failed to reduce the severity of visible metastases (Figure 3f, Table S1) or organ weights (Figure 3g,h). Interestingly, while the drug combination did not have a significant effect on organ weight, it did reduce the severity of lesions, compared to vehicle or sunitinib alone (Figure 1f–h, Table S1).

### 2.4. SLC-0111 Reduces Liver Metastatic Burden

To investigate in more detail the impact of SLC-0111 and sunitinib—alone and in combination—on the extent of liver metastases, we stained whole mount liver tissue slices by immunofluoresence for vimentin to detect human tumor cells. Fluorescently-labeled dextran was also imaged to allow visualization of overall liver sinusoid architecture. Liver tissue from animals administered SLC-0111, but not those exposed to sunitinib, showed markedly reduced staining for vimentin, compared to vehicle control (Figure 4a). Furthermore, reduced staining for vimentin was clearly evident in livers from animals administered sunitinib and SLC-0111 in combination, relative to either sunitinib alone or vehicle (Figure 4a). Quantification revealed a significant reduction in vimentin-positive area in livers from mice given SLC-0111, both alone and in combination with sunitinib (Figure 4b), demonstrating that treatment with SLC-0111 is effective at reducing liver metastasis.

Next, we determined whether CAIX is expressed by liver metastases and whether drug administration impacts CAIX expression. We observed that liver metastases express CAIX, and that while SLC-0111 alone did not impact CAIX expression, sunitinib dramatically increased the levels of CAIX expressed by these metastases (Figure 4c). The combination of SLC-0111 with sunitinib markedly reduced the level of CAIX expression, compared to sunitinib alone (Figure 4c). Quantification confirmed that sunitinib significantly increased CAIX expression by liver metastases and that the sunitinib-induced increase could be abrogated by SLC-0111 (Figure 4d).

### 2.5. Administration of SLC-0111 Alone and in Combination with Sunitinib Reduces Lung Metastases

**Figure 5:**
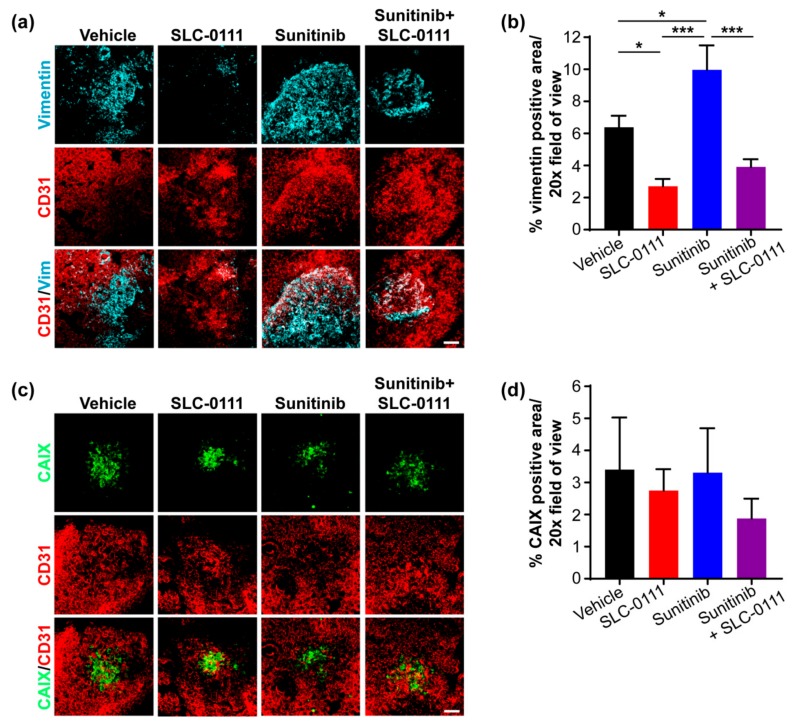
Administration of SLC-0111 reduces lung metastases. (**a**) Representative 3D maximum projections of whole mount lung tissue slices from tumor-bearing mice, showing vimentin-positive metastases (cyan) and CD31^+^ blood vessels (red). Scale bar = 100 μm. (**b**) Analysis of vimentin positive metastases in whole mount lung tissue sections described in panel a. Data show the mean ± standard error of the mean (SEM). *n* = 8–10 images/group. * *p* < 0.05, *** *p* < 0.001. (**c**) Representative 3D maximum projections of whole mount lung tissue slices from tumor-bearing mice, showing Carbonic Anhydrase IX (CAIX)-positive metastases (green) and CD31^+^ blood vessels (red). Bar = 100 μm. (**d**) Analysis of CAIX positive metastases in the whole mount lung tissue sections described in panel c. Data show the mean ± SEM. *n* = 5–8 images/group: No statistically significant differences were observed among the groups.

Next, vimentin staining was carried out on whole mount lung tissue preparations to detect and evaluate metastases within the lung microenvironment. Consistent with our findings showing the relative absence of lung surface nodules in several mice administered SLC-0111 (see Figure 3, Table S1), staining for vimentin was significantly reduced in the lungs of animals given the inhibitor (Figure 5a,b). In sharp contrast, sunitinib clearly increased vimentin staining compared to vehicle controls, while the administration of sunitinib and SLC-0111 in combination significantly reduced vimentin-positive foci in the lungs, compared to sunitinib treatment alone (Figure 5a,b).

The metastatic foci in the lungs were also evaluated for levels of CAIX expression. While CAIX expression was observed across all treatment groups (Figure 5c), there was no significant difference amongst the four groups (Figure 5d), in contrast to the treatment-defined levels of CAIX expression observed in the liver metastases. It is possible that the oxygenated microenvironment present in the lungs, as demonstrated by CD31 staining (Figure 5c,d), functions to restrict the levels of CAIX present in this tissue.

## 3. Discussion

TNBC is aggressive, metastatic and drug-resistant, limiting the spectrum of effective therapeutic options for breast cancer patients [3,4]. Anti-angiogenic agents have had limited success in the treatment of systemic disease in breast cancer, possibly due to exacerbation of hypoxia and increased metastasis. Here, using a highly metastatic, in vivo model of CAIX-positive human TNBC, we demonstrate that the administration of a clinically validated inhibitor of CAIX activity, SLC-0111, dramatically reduces spontaneous metastases, whereas the decrease in tumor growth, while evident, is more modest, suggesting that the reduction in metastasis observed with inhibition of CAIX is not due solely to reduced tumor growth. In contrast, the administration of the anti-angiogenic tyrosine kinase inhibitor, sunitinib, significantly reduces primary tumor growth, but results in exacerbation of tumor hypoxia and increased CAIX expression by tumor cells, culminating in increased metastasis. Importantly, the combination of sunitinib and SLC-0111 inhibits both primary tumor growth and distant metastases, thereby effectively controlling local and systemic disease, providing an innovative potential therapeutic strategy for TNBC.

This is the first study to demonstrate that the pharmacologic targeting of CAIX using a highly selective inhibitor in combination with anti-angiogenic agents results in both a significant reduction in tumor growth as well as marked inhibition of distant metastasis. While previous studies have reported that sunitinib can increase hypoxia and metastasis in TNBC [5,6], our findings clearly show that CAIX expression is increased in the primary tumor and metastases in response to sunitinib exposure, and that selectively targeting CAIX in this context results in increased efficacy. Our findings are supported by studies showing that the genetic depletion of CAIX expression enhances the effect of the anti-angiogenic agent bevacizumab on tumor growth in a preclinical tumor model of glioblastoma [29]. In addition, treatment of the cholangiocarcinoma with acetazolamide, a pan CAIX inhibitor, increased the growth-inhibitory effects of bevacizumab [33].

**Figure 6:**
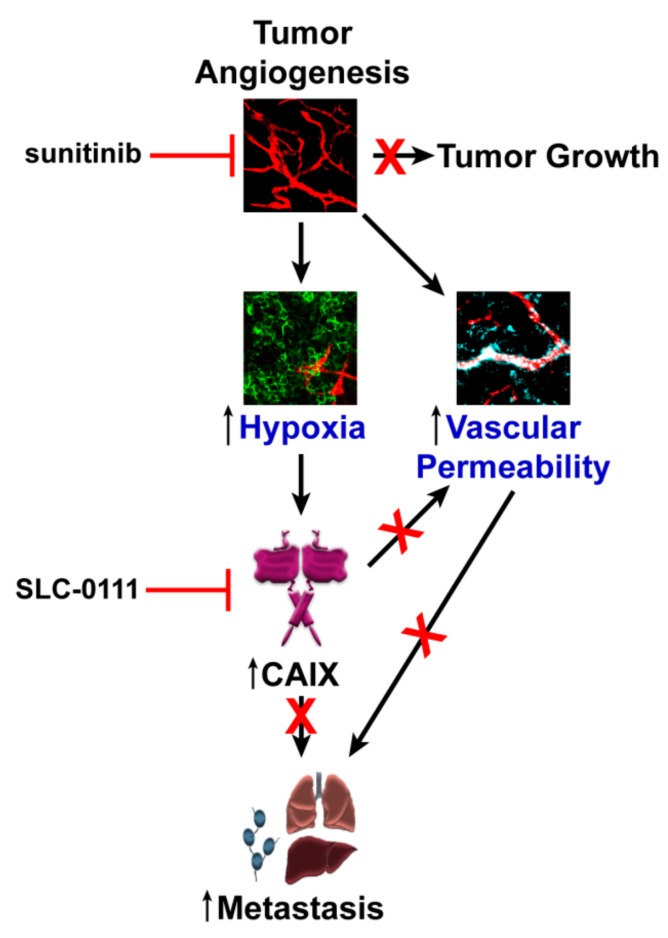
Model for targeting angiogenesis and Carbonic Anhydrase IX (CAIX) to reduce metastasis of Triple Negative Breast Cancer (TNBC). Exposure of tumors to anti-angiogenic agents such as sunitinib leads to reduced tumor growth. However, major consequences can include enhanced hypoxia and increased vascular permeability. The exacerbation of hypoxia results in the upregulation of CAIX expression by tumor cells and the potentiation of metastasis. Inhibition of CAIX activity inhibits metastasis through several mechanisms, as shown by previous studies, but may also play a role in reducing vascular permeability and contributing to vascular normalization, potentially reducing the invasion of tumor cells to distant sites.

Our findings show that a major result of CAIX blockade is a reduction of not only the number of blood vessels in the primary tumor, but also of the permeability of the remaining vasculature (Figure 6). These data suggest that inhibition of CAIX activity may be involved the promotion of vessel normalization, a process that involves initial pruning of immature vessels, followed by fortification and reduced permeability of the remaining vasculature [7]. Anti-angiogenic agents themselves are known to result in vessel normalization in a subset of patients, but risks include excessive pruning and rebound angiogenesis if the pre-treatment vasculature is not of sufficient density, resulting in increased hypoxia [7]. A significant clinical challenge currently is the prospective identification of patients who will respond to anti-angiogenics with vessel normalization. In this study, the administration of sunitinib reduced the number of blood vessels and increased vascular permeability, while SLC-0111 significantly reduced the sunitinib-induced increase in permeability, suggesting that SLC-0111 may be capable of promoting increased vascular fitness, even in situations of low vessel density, potentially enabling the use of anti-angiogenics for a diverse breast cancer patient population.

While the exact mechanism by which CAIX inhibition may promote vascular normalization remains to be elucidated, the normalized vasculature observed within tumors treated with SLC-0111 may be, in part, a consequence of reduced levels of angiogenic factors present in the tumor microenvironment. Our data showing that genetic depletion of hypoxia-induced CAIX expression by MDA-MB-231 LM2-4Luc+ cells in vitro results in reduced levels of VEGFA suggests that decreased VEGFA production may be a potential mechanism for the effects of SLC-0111 on normalization of tumor vasculature. Alternatively, recent studies have shown that CAIX-containing exosomes released by hypoxic renal cell carcinoma cells are taken up by HUVEC and promote migration and tube formation by human umbilical vein endothelial cells (HUVEC) in angiogenesis assays in vitro [34], raising the interesting possibility that CAIX-positive tumor cells may give rise to CAIX-containing exosomes in the tumor microenvironment of TNBC, offering an additional CAIX positive target for inhibition.

While we have not directly assessed the levels of specific pro-metastatic chemokines and their cognate receptors, such as CXCR4, in the present study, previous work has demonstrated that CAIX is required for nuclear factor kappa-light-chain-enhancer of activated B cells (NF-κB)-mediated production of granulocyte colony stimulating factor (G-CSF) by hypoxic breast cancer cells in vivo, and that CAIX is required for the G-CSF-driven mobilization of granulocytic myeloid-derived suppressor cells (MDSCs) to the breast cancer lung metastatic niche [35]. These data suggest that inhibition of can result in altered production of soluble mediators that negatively impact the metastatic niche, culminating in reduced metastasis. Furthermore, it has recently been shown that CAIX interacts with matrix metalloprotease (MMP)-14, and activates it at the plasma membrane by contributing protons [36]. The increased MMP-14 activity results in increased extracellular matrix (ECM) (especially collagen) degradation. However, while CAIX activity contributes to the extracellular accumulation of protons and contributes to extracellular acidosis, the direct effects of CAIX inhibition on specific, collagen-based fibrotic markers in breast cancer in vivo remain to be determined.

Importantly, the reduced permeability resulting from exposure to SLC-0111 has several potentially favorable consequences. A more functional vasculature would potentially result in the more efficient delivery of drug therapy, together with a reduction in hypoxia and a less hostile TME. Furthermore, reduced permeability would consequently lead to less intravasation, reducing the capacity of tumor cells to escape and seed distant sites. Whether this effect of CAIX inhibition is due to direct effects on the tumor cells (i.e., on down-regulation of angiogenic factors or up-regulation of endogenous angiogenesis inhibitors) or occurs via regulation of the TME needs to be further explored using genetic gain- or loss-of-function models.

Our data also demonstrate that CAIX is expressed by breast tumor cells resident both in liver and lung metastases, demonstrating that these cells maintain target expression in the metastatic niche, and providing an opportunity for inhibiting cells at the primary tumor site, as well as in metastases. Exposure to sunitinib increased CAIX expression in the liver, while the administration of SLC-0111, both alone and in combination with sunitinib, markedly reduced liver metastatic burden, suggesting that SLC-0111 is likely acting directly on liver metastatic cells. In the lung, sunitinib significantly increased metastatic burden, while SLC-0111 alone, and in combination with sunitinib, reduced metastases. However, CAIX expression in the lung does not change regardless of treatment, possibly due to exposure of the lung vasculature to high levels of oxygen. Thus, control of lung metastasis in this model may lie in the upstream effects of SLC-0111 on vascular permeability of the primary tumor. These results also indicate the potential utility of targeting CAIX in cancer patients, since inhibition would both target the primary tumor and metastases.

In conclusion, we have demonstrated that the use of specific pharmacologic inhibitors of CAIX activity in combination with anti-angiogenic agents results in inhibition of tumor growth and reduced lung metastasis in TNBC. Our findings suggest that targeting angiogenesis and hypoxia effectors in combination holds promise as a novel rational strategy for the effective treatment of patients with TNBC.

## 4. Materials and Methods

### 4.1. Cells

MDA-MB-231 LM2-4Luc^+^ human TNBC cells [37], which are highly metastatic and show preferred tropism to the lung, were kindly provided by Dr. Robert Kerbel (University of Toronto, ON, Canada). The MDA-MB-231 LM2-4Luc^+^ human TNBC cell lines stably depleted of CAIX by shRNA have been described previously [21]. Cells were maintained in phenol red-free Roswell Park Memorial Institute (RPMI) media (11835-030; ThermoFisher Scientific, Burlington, ON, Canada), supplemented with 10% fetal bovine serum (FBS) (12483-020; ThermoFisher Scientific, Burlington, ON, Canada). Stocks of cells were stored in liquid nitrogen, cultured in a humidified incubator at 37 °C with 5% CO_2_ and ambient O_2_, and passaged a maximum of 5 times prior to inoculation into animals. For studies in hypoxia, cells were grown at 37 °C in an atmosphere of 1% O_2_, 5% CO_2_, 94% N_2_ in a humidified incubator inside a sealed workstation as described previously [21]. The cells used in the manuscript were authenticated using short tandem repeat DNA profiling (DNA fingerprinting) by a commercial testing facility (Genetica, Burlington, NC, USA) and were found to exhibit 92% identity when compared to ATCC-derived wildtype MDA-MB-231 human breast cancer cells.

### 4.2. Animal Studies

All animal studies and associated procedures were carried out in accordance with protocol A14-0058 approved by the Institutional Animal Care Committee at the British Columbia Cancer Research Centre and the University of British Columbia (Vancouver, BC, Canada), with adherence to the guidelines set out by the Canadian Council on Animal Care (CCAC) (approval date: 1 March 2014). MDA-MB-231 LM2-4Luc^+^ cells (2 × 10^6^ cells/animal) were orthotopically implanted into female NOD.SCID IL-2R knockout (NSG) or NOD.SCID (NSD) mice as described previously [19]. Animals bearing established tumors (average tumor volume of 130–150 mm^3^) were randomly divided into groups and administered SLC-0111 alone, sunitinib alone, sunitinib and SLC-0111 in combination or vehicle. The administration of SLC-0111 was performed by oral gavage daily at a concentration of 50 mg/kg, as previously described [19]. Sunitinib (S-8803; LC Laboratories, Woburn, MA, USA) was administered by oral gavage daily at the dose of 60 mg/kg as described previously [5]. At the experimental endpoint, mice were euthanized and primary tumors, all visible metastases, lungs and livers were harvested. In some experiments, mice were administered, by intraperitoneal (i.p.) injection, bromodeoxyuridine (BrdU) (1.5 mg/kg, B5002-5G; Sigma-Aldrich, Oakville, ON, Canada) 120 minutes prior to euthanasia and pimonidazole (60 mg/kg, HP2-100Kit, Hypoxyprobe, Burlington, MA, USA) 60 minutes prior to euthanasia to detect proliferation and hypoxia, respectively. For experiments assessing vascular permeability, lysine-fixable tetramethylrhodamine dextran (LRD) with a molecular weight of 70 kDa (100 µL, D1818; ThermoFisher Scientific, Burlington, ON, Canada) was injected into the tail vein of tumor-bearing mice 5 min prior to euthanasia, as previously described [38,39]. Harvested tissues were weighed, photographed and placed in 4% paraformaldehyde (PFA; 158127, Sigma-Alrich, Oakville, ON, Canada) or formalin (3800604EG, Leica Biosystems, Concord, ON, Canada) for downstream processing and analysis.

### 4.3. Tumor Growth and Metastasis

To monitor primary tumor growth, tumors were measured 3x/week using digital calipers and tumor volumes were calculated using the modified ellipsoid formula, (L × W2)/2 (L, length; W, width). Metastasis was monitored once per week by bioluminescence imaging (BLI) using an In Vivo Imaging System 200 (IVIS 200; PerkinElmer, Waltham, MA, USA) as previously described [19]. Briefly, 200 μL/mouse of D-luciferin (15 mg/mL; 122799; Perkin Elmer, Waltham, MA, USA) was administered by i.p. injection, animals were held for 10 minutes and subsequently imaged under isoflurane anesthesia. Metastasis was quantified by calculating the Total Flux of photons using Living Image Software (Perkin-Elmer, Waltham, MA, USA).

### 4.4. Histochemistry and Immunohistochemistry (IHC)

Formalin-fixed, paraffin-embedded (FFPE) tissues were sectioned (4–5 μm) and stained with primary antibodies against human CAIX (1:50, AF2188; R&D Systems, Minneapolis, MN, USA), CD31 (1:10, DIA-310; Histobiotec, Miami Beach, FL, USA), BrdU (1:10, Roche, Laval, QC, Canada) and pimonidazole (1:100, Hydroxyprobe, Burlington, MA, USA). For IHC, ImmPRESS™ HRP Anti-Rat IgG (MP-7444; Vector Laboratories, Burlingame, CA, USA), HRP Anti-Goat IgG (MP-7405; Vector Laboratories, Burlingame, CA, USA), and HRP Anti-Mouse/Rabbit IgG (MP-7500; Vector Laboratories, Burlingame, CA, USA) secondary antibodies were used according to the manufacturer’s instructions and signal was detected using a 3,3’-diaminobenzidine (DAB) horseradish peroxidase (HRP) Substrate Kit (SK-4100; Vector Laboratories, Burlingame, CA, USA) as previously described [19]. For each marker, at least 10 randomly selected fields of view at a magnification of 20x were imaged from 1 tissue section/tumor and 3 tumors were analyzed for each group. The percent area of positive staining in each image was quantified using ImageJ (v1.48, National Institutes of Health, Bethesda, MD, USA).

### 4.5. Whole Mount Staining

Tissues were fixed in 4% paraformaldehyde (PFA) overnight and then sliced thinly by hand using a scalpel. Care was taken to select geographically similar areas from each tumor and to avoid areas of excessive necrosis. Thus, tissues were first bisected and then further sectioned on the diagonal approximately 25% of the way from the tumor margin, at which point thin slices were cut for staining.

Tissue slices were washed in PBS and digested with Proteinase K (20μg/mL, 25530049; Invitrogen Life Technologies, Burlington, ON, Canada) for 5 min at room temperature (RT). Tissues were then post-fixed in methanol for 30 min, washed in PBS for 30 min and incubated overnight (O/N) in blocking buffer (3% milk in 0.3% Triton-X-100 PBS) at 4 °C. Subsequently, tissues were incubated with primary antibodies diluted to appropriate concentrations in buffer (0.3% Triton-X-100 PBS) O/N at 4 °C. Primary antibodies included CD31 (1:200 dilution, 550274; BD Biosciences, Mississauga, ON, Canada), CAIX (1:200, AF2188; R&D Systems, Minneapolis, MN, USA) and vimentin (1:200 dilutions, 550513; BD Biosciences, Mississauga, ON, Canada). The following day, tissues were washed in PBS for 1.5 h at 4 °C and incubated with blocking buffer for 1.5 h at RT. Appropriate secondary antibodies diluted in blocking solution were then applied [Donkey anti-Goat Alexa 488 (705-545-147; Jackson Immunoresearch, West Grove, PA, USA), Donkey anti-Rabbit Cy5 (711-175-152; Jackson Immunoresearch, West Grove, PA, USA or A11007; Invitrogen Life Technologies, Burlington, ON, Canada), or Goat anti-Rat Alexa 555 (A-21434; Invitrogen Life Technologies, Burlington, ON, Canada)] and tissues were incubated at 4 °C for 2 h. Subsequently, tissues were washed and incubated in modified blocking buffer (0.15% blocking solution) for 1 h at 4 °C, followed by a solution change and continued incubation in modified blocking buffer O/N at 4 °C. Finally, tissues were mounted with Vectashield mounting medium (H-1000; Vector Laboratories, Burlingame, CA, USA).

### 4.6. Imaging and Quantification of Whole Mount Immunostained Tissue Slices

For the imaging of whole mount tissue slices, Z stacks were acquired from randomly chosen fields of view using a Nikon Ti confocal microscope (Nikon, Mississauga, ON, Canada) equipped with a 20x objective lens. A 3D maximum projection was generated for each Z stack for downstream analysis and quantification.

The percent area of each image positively stained for a given marker was quantified using ImageJ or Adobe Photoshop CS5 (Adobe Systems Inc, San Jose, CA, USA). 3D maximum projection images were used for quantification. CD31^+^ blood vessels were enumerated using the cell counter plugin for ImageJ. For the purposes of analysis, each side branch stemming from a larger vascular structure was counted as an individual vessel. The amount of extravasated dextran was determined by subtracting the total dextran signal in a given image from the dextran signal colocalizing with staining for CD31 using Photoshop. For each quantified parameter, the number of images analyzed for each group is reported in the appropriate Figure legend.

### 4.7. Analysis of Vascular Permeability

Tissues labeled in vivo with Lysine-fixable tetramethylrhodamine dextran (LRD) having a molecular weight of 70 kDa were whole mount immunostained as described above with antibodies targeting CD31 (1:200, 550274; BD Biosciences, Mississauga, ON, Canada) for detection of tumor blood vessels. Tissue slices were imaged as Z stacks by a Nikon Ti confocal microscope equipped with a 20x objective lens (Nikon, Mississauga, ON, Canada) and 3D maximum projections were produced for downstream analysis. Extravasation of LRD was quantified from randomized fields (20x magnification) of each group using ImageJ software (v1.48, National Institutes of Health, Bethesda, MD, USA).

### 4.8. Analysis of Angiogenesis-Related Proteins

Conditioned media collected from the indicated cell lines was analyzed using the Human Angiogenesis Antibody Array (AY007; R&D Systems, Minneapolis, MN, USA) according to the manufacturer’s instructions. Positive signals were quantified using ImageJ software (v1.48, National Institutes of Health, Bethesda, MD, USA). The mean signal of duplicate spots representing each angiogenesis-related protein was normalized to the mean value of the reference signals present on the array.

### 4.9. Quantitative real time PCR (qPCR)

qPCR for CAIX and VEGFA expression was carried out as previously described [35]. Briefly, RNA was extracted from hypoxic MDA-MB-231LM2/4 cells expressing non-silencing shRNA (shNS) and shRNA targeting CAIX (shCAIX) using the RNeasy plus mini kit (Qiagen, Germantown, MD, USA) and quantified by UV-Vis spectroscopy. RNA (500 ng) was reverse transcribed into cDNA using the Superscript III first-strand synthesis kit (Invitrogen Life Technologies, Burlington, ON, Canada) as per the manufacturer’s instructions. Amplification of the DNA was performed using FAST SYBR Green Master mix real-time qPCR on an Applied Biosystems 7900HT (Foster City, CA, USA). Quantification of the levels of the individual genes was done using the formula q = E−CT, where E represents the primer efficiency and CT the threshold cycle. Expression of each gene was normalized to β-actin mRNA levels and data are expressed as fold change relative to the shNS sample.

### 4.10. Statistical Analysis

Statistical analyses were performed using GraphPad Prism 7 (GraphPad Software, Inc, San Diego, CA, USA). Outliers were identified using Grubb’s test (alpha = 0.05) and excluded from further analysis. Data are reported as the mean ± standard error of the mean (SEM). For comparison of three or more data sets, a one-way analysis of variance (ANOVA) was performed and a Holms-Sidak’s test was used to correct of multiple comparisons. Statistical significance was set at *p* < 0.05. 

## 5. Conclusions

In conclusion, we have generated robust data demonstrating that the use of pharmacologic inhibitors of CAIX activity in combination with anti-angiogenic agents results in the inhibition of tumor growth and reduced lung metastasis in TNBC. Our findings suggest that targeting angiogenesis and hypoxia in combination holds promise as a novel rational strategy for the effective treatment of patients with TNBC.

## Supplementary Materials

The following are available online at https://www.mdpi.com/2072-6694/11/7/1002/s1, Table S1: Number of animals with grossly visible metastasis in lungs, Figure S1: Representative images of MDA-MB-231/LM2-4Luc+ primary tumor tissue sections from animals administered SLC-0111 and sunitinib, alone and in combination, and immunohistochemically stained for Ki67, Figure S2: Mean body weights of animals administered Sunitinib and SLC-0111, Figure S3. Short hairpin RNA (shRNA)-mediated knockdown of Carbonic Anhydrase IX (CAIX) reduces expression of angiogenic factors by breast cancer cells in hypoxia.
