# Supplementary material for: Harnessing Induced Essentiality: Targeting Carbonic Anhydrase IX and Angiogenesis Reduces Lung Metastasis of Triple Negative Breast Cancer Xenografts
Source: Cancers (Basel). 2019 Jul 17;11(7):1002. doi: 10.3390/cancers11071002 (PMC6678951; doi:10.3390/cancers11071002)
Supplement: Supplementary file 1 [file cancers-11-01002-s001.pdf]

Supplementary Materials

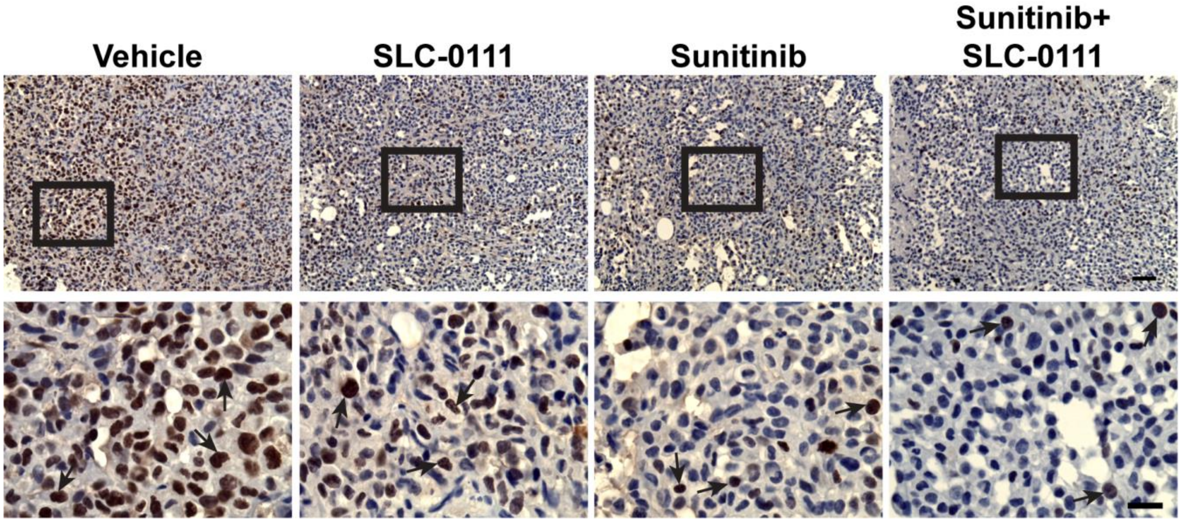

Figure S1. Representative images of MDA-MB-231/LM2-4Luc+ primary tumor tissue sections from animals administered SLC-0111 and sunitinib, alone and in combination, and immunohistochemically stained for Ki67 (arrows). Boxes indicate regions of interest shown at higher magnification in the lower panels. Scale bars: upper panels, 100 μm; lower panels, 20 μm.

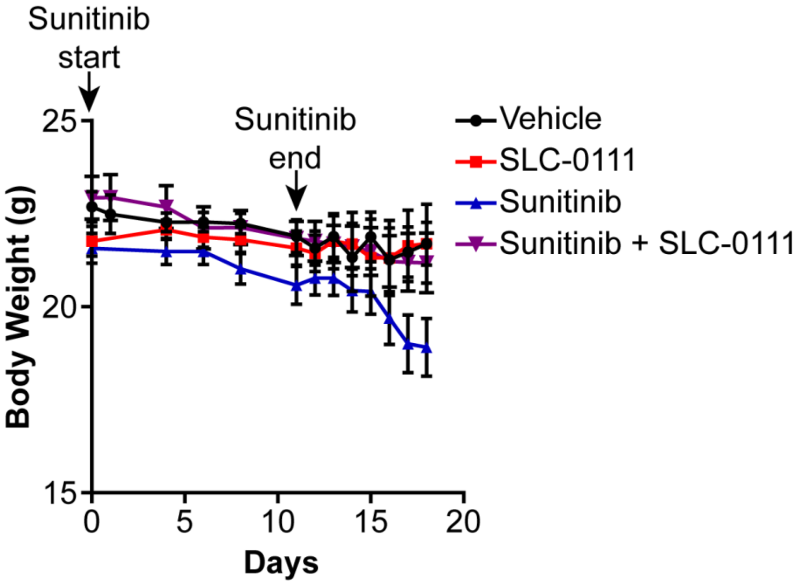

Figure S2. Mean body weights of animals administered Sunitinib and SLC-0111. Initiation and completion of dosing of sunitinib are indicated by the arrows. SLC-0111 was administered daily until endpoint. Data show the mean ± standard error of the mean (SEM). *n* = 8–10 animals/group.

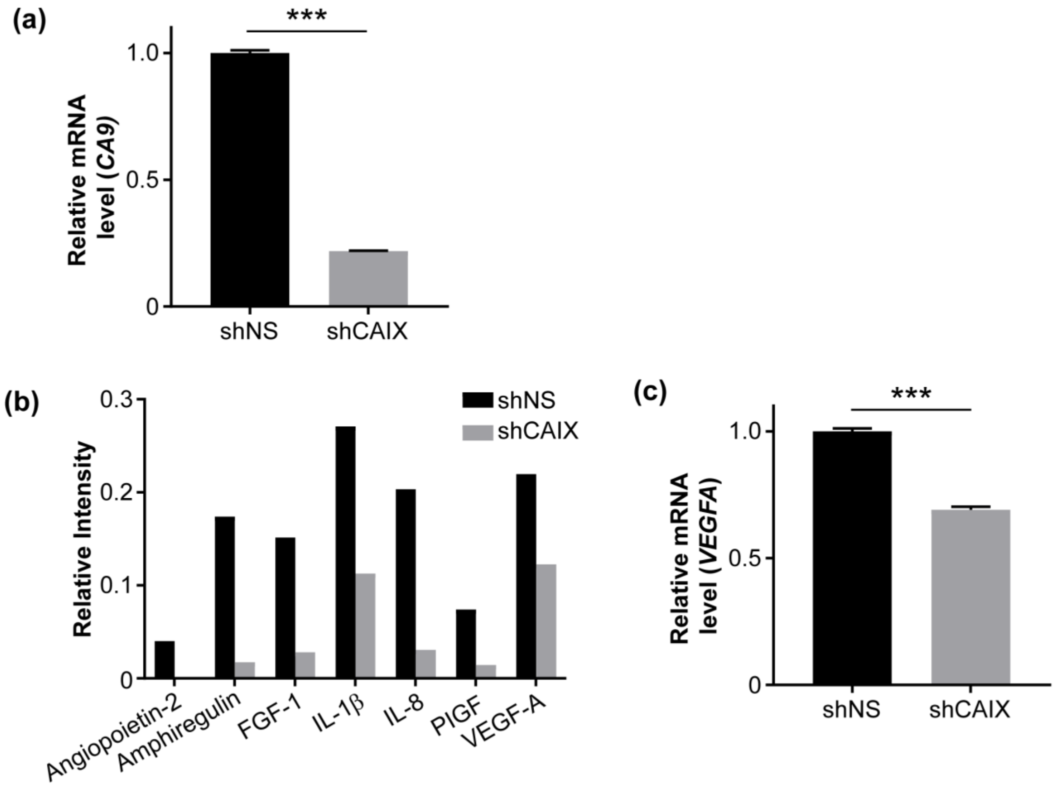

Figure S3. Short hairpin RNA (shRNA)-mediated knockdown of Carbonic Anhydrase IX (CAIX) reduces expression of angiogenic factors by breast cancer cells in hypoxia. (**a**) MDA-MB-231LM2 cells expressing shRNA targeting CAIX were cultured in hypoxia for 72 hours and analyzed for Carbonic Anhydrase 9 (CA9) mRNA levels by qPCR. (**b**) Media conditioned by MDA-MB-231LM2 cells depleted of CAIX and cultured for 72 hours in hypoxia were analyzed for several angiogenesis-related proteins using a human angiogenesis antibody array. The mean normalized relative intensity of duplicate samples is shown. (**c**) MDA-MB-231LM2 cells expressing shRNA targeting CAIX were cultured in hypoxia for 72 hours and analyzed for Vascular Endothelial Growth Factor A (VEGFA) mRNA levels. *** $p < 0.001$.

Table S1. Number of animals with grossly visible metastasis in lungs.

	Extensive Metastasis	Small Visible Metastatic Foci	No Metastasis
**Vehicle**	4/5	1/5	0/5
**SLC-0111**	3/8	2/8	3/8
**Sunitinib**	6/6	0/6	0/6
**Sunitinib + SLC-0111**	6/7	1/7	0/7
